# Value of saliva testing when added to questionnaire screening for unhealthy drug use

**DOI:** 10.1186/1940-0640-10-S2-O25

**Published:** 2015-09-24

**Authors:** H John Saade, Anthony Dedea, Lauren Dedea, Jason Dhabliwala, Andrea T Pusser, J Aaron Johnson, J Paul Seale

**Affiliations:** 1 Department of Family Medicine, Navicent Health/Mercer University School of Medicine, Macon, GA, USA; 2 Institute of Public and Preventive Health, Georgia Regents University, Augusta, GA, USA

## Background

Unhealthy drug use (UDU), including both illicit drug use and misuse of prescription medications, is high among Americans 12 or older (9.4% past month; 48% lifetime), and is often 50-100% higher among emergency department (ED) patients (Cherpitel & Ye, 2008). While most screening, brief intervention and referral to treatment (SBIRT) projects focus on questionnaire screening for UDU, many clinicians are unaware of the potential of saliva testing (ST) to increase detection.

The objective is toassess the added value of ST when added to comprehensive questionnaire screening for UDU.

## Material and methods

Research assistants systematically approached adult patients receiving care in the medical-surgical area of an urban ED. After granting informed consent, patients completed a survey containing four different single-item drug screening questions (SDSQs) and the drug use section of the Mini International Neuropsychiatric Interview, then received a financial incentive. Patients granting separate consent then provided a saliva sample for drug testing and received a second incentive.

## Results

Among 208 patients interviewed, only 111 (53.4%) agreed to saliva testing. Nine samples were positive in patients not reporting illegal/recreational drug use. Six were positive for prescribed medications which they had reported (five for alprazolam, one for opioids). Two were positive for undisclosed cocaine. One was positive for barbiturates in a patient who did not report barbiturate use.

## Conclusions

ST detected two patients (1.8% of those submitting samples) with unsuspected illicit drug use and one patient (0.9%) with unreported prescription psychotropic drug use. Despite financial incentives, 47% of patients refused ST, which could have disclosed more unreported drug use. Results confirm the ability of biomarker testing to detect small numbers of additional patients with potentially life-threatening UDU when added to questionnaire screening.
